# Working with Zika and Usutu Viruses *In Vitro*

**DOI:** 10.1371/journal.pntd.0004931

**Published:** 2016-08-19

**Authors:** Kelli L. Barr, Benjamin D. Anderson, Dhani Prakoso, Maureen T. Long

**Affiliations:** 1 Department of Infectious Diseases & Pathology, College of Veterinary Medicine, University of Florida, Gainesville, Florida, United States of America; 2 Division of Infectious Disease, School of Medicine and Global Health Institute, Duke University, Durham, North Carolina, United States of America; Centers for Disease Control and Prevention, UNITED STATES

## Abstract

Usutu (USUV) and Zika (ZIKV) viruses are emerging arboviruses of significant medical and veterinary importance. These viruses have not been studied as well as other medically important arboviruses such as West Nile (WNV), dengue (DENV), or chikungunya (CHIKV) viruses. As such, information regarding the behavior of ZIKV and USUV viruses in the laboratory is dated. Usutu virus re-emerged in Austria in 2001 and has since spread throughout the European and Asian continents causing significant mortality among birds. Zika virus has recently appeared in the Western Hemisphere and has exhibited high rates of birth defects and sexual transmission. Information about the characteristics of USUV and ZIKV viruses are needed to better understand the transmission, dispersal, and adaptation of these viruses in new environments. Since their initial characterization in the middle of last century, technologies and reagents have been developed that could enhance our abilities to study these pathogens. Currently, standard laboratory methods for these viruses are limited to 2–3 cell lines and many assays take several days to generate meaningful data. The goal of this study was to characterize these viruses in cells from multiple diverse species. Cell lines from 17 species were permissive to both ZIKV and USUV. These viruses were able to replicate to significant titers in most of the cell lines tested. Moreover, cytopathic effects were observed in 8 of the cell lines tested. These data indicate that a variety of cell lines can be used to study ZIKV and USUV infection and may provide an updated foundation for the study of host-pathogen interactions, model development, and the development of therapeutics.

## Author Summary

Usutu and Zika viruses are arboviruses of significant medical and veterinary outbreaks in recent years. Currently, standard laboratory methods for these viruses are limited to 2–3 cell lines. Here, our studies demonstrate that Zika and Usutu viruses are able to replicate in cells from a wide range of animal cell lines. The data will allow for further study of the potential for evolution of these viruses in other hosts.

## Introduction

Usutu virus (USUV), first identified in South Africa in 1959, is a flavivirus belonging to the Japanese encephalitis complex [1,2]. In 2001, USUV emerged in Austria and spread throughout the European and Asian continents [3–10]. Unlike USUV circulating in Africa, the new emergent strains caused significant mortality among European blackbirds, owls, and other wild and captive birds [3,11]. The life cycle of USUV is composed of transmission from primarily *Culex* mosquito vectors to avian reservoir hosts in a sylvatic transmission cycle [1]. Other than birds, evidence for USUV infection has been found in humans, horses, and bats [12–15]. Several human cases have been identified in Europe and Croatia [16–18]. Recently, USUV has been linked to neuroinvasive infections in 3 patents from Croatia [10] and has been detected in horses in Tunisia [14].

Zika virus (ZIKV) is an emerging, medically important arbovirus. There are two geographically distinct lineages of circulating ZIKV; African and Asian [19]. The Asian lineage has recently emerged in Micronesia where it was the cause of a large outbreak in 2007 [20] and currently in the Americas [21]. The natural hosts of ZIKV include humans, primates, and *Aedes* mosquitos [22–25]. Though no solid evidence exists of non-primate reservoirs of ZIKV [26], antibodies to ZIKV have been detected in elephants, goats, lions, sheep, zebra, wildebeests, hippopotamuses, rodents, and other African ruminants [27,28]. Like many other tropical arboviruses, human infection with ZIKV typically presents as either asymptomatic or acute febrile illness with fever, rash, headache, and myalgia. The flavivirus, dengue virus (DENV) and the alphavirus, chikungunya virus (CHIKV) produce similar symptoms to ZIKV but are more commonly diagnosed. The high seroprevelance of ZIKV antibodies in human populations in Africa and Asia suggests the misdiagnosis of ZIKV for other arboviral illnesses is an ongoing problem [19].

There are several characteristics of ZIKV that distinguish it from other medically important arboviruses. In recent outbreaks, ZIKV has exhibited atypical symptoms including respiratory involvement and frequent conjunctivitis [20,29]. ZIKV also has the ability to spread from human to human through sexual and maternal-fetal transmission [30–32]. ZIKV has been linked to serious medical conditions such as microcephaly and other brain abnormalities in neonates and Guillain-Barré (GB) syndrome in adults [31–33].

While research in serology and genetic characterization are underway [19,20], the recent changes in biology and distribution of these viruses warrant further investigation as many questions regarding the basic biology and ecology of ZIKV and USUV remain unanswered. To better understand the characteristics of USUV and ZIKV *in vitro*, we investigated the permissiveness of several cell lines.

## Materials and Methods

### Cells and viruses

Seventeen cell lines were obtained from the ATCC (Manassas, VA) and included Tb 1 Lu, DF-1, Sf 1 Ep, EA.hy.926, CRFK, E.Derm, FoLu, Pl 1 Ut, OHH1.K, OK, DN1.Tr, PK(15), LLC-MK2, BT, MDCK, WCH-17, and Mv 1 Lu (Table 1). These lines were selected to include domestic and peridomestic representatives of species found only in the Americas; specifically, North America. All cell lines were passaged 5 times after the initial expansion from the ATCC stock prior to experiments. All cells were cultured in Dulbecco's modified eagle medium (DMEM) supplemented with 10% (v/v) fetal bovine serum (FBS), 4mM L-glutamine, 10 mM non-essential amino acids (NEAA), 1 mM sodium pyruvate, 100 U/ml penicillin, 100 μg/ml streptomycin, and housed in a 37°C incubator with 5% CO2.

**Table 1:** Cell lines used for characterization of USUV and ZIKV.

Cell Line	Common Name	Species	Tissue	ATCC No.
Tb 1 Lu	Free-tailed bat	Tadarida brasiliensis*	Lung epithelial	CCL-88
DF-1	Chicken	*Gallus gallus*	Embryonic fibroblast	CRL-12203
Sf 1 Ep	Cottontail rabbit	Sylvilagus floridanus*	Epidermis epithelial	CCL-68
EA.hy.926	Human	*Homo sapiens*	Vascular endothelial	CRL-2922
CRFK	Domestic cat	*Felis catus*	Kidney epithelial	CCL-94
E.Derm	Horse	*Equus caballus*	Dermis fibroblast	CCL-57
FoLu	Grey fox	Urocyon cineroargenteus*	Lung fibroblast	CCL-168
Pl 1 Ut	Raccoon	*Procyon lotor*	Uterus fibroblast	CCL-74
OHH1.K	Mule deer*	Odocoileus hemionus*	Kidney fibroblast	CRL-6193
OK	Virginia opossum	Didelphis virginiana*	Kidney epithelial	CRL-1840
DNl.Tr	Nine-banded armadillo	Dasypus novemcinctus*	Trachea fibroblast	CRL-6009
PK(15)	Domestic pig	*Sus scrofa*	Kidney epithelial	CCL-33
LLC-MK2	Rhesus monkey	*Macaca mulatta*	Kidney epithelial	CCL-7
BT	Cow	*Bos taurus*	Turbinate	CRL-1390
MDCK	Domestic dog	*Canis familiaris*	Kidney epithelial	CCL-34
WCH-17	Eastern woodchuck	Marmota monax*	Liver epithelial	CRL-2082
Mv 1 Lu	American mink	Neovison vison*	Lung epithelial	CCL-64

*Indicates that species is native to the Western Hemisphere.

USUV (SAAR-1776), ZIKV (MR766 –original, African), YFV (17D), Sindbis virus (SINV EgAr 339), CHIKV (181/25), DENV-1 (H87), DENV-2 (NGC), DENV-3 (HI), and DENV-4 (H241) were obtained from the World Reference Center for Emerging Viruses and Arboviruses (Robert Tesh, UTMB, Galveston, TX). These viruses were of low passage stock that had been in storage since the mid-20^th^ century. ZIKV (PRVABC59 Puerto Rico 2015, Asian) was obtained from the American Tissue Type Collection and RNA was extracted directly from the sample upon arrival. WNV (NY99) was obtained in 2001 from the National Veterinarian Services Laboratory and had undergone only 2 expansions prior to use. The titers of all viruses were determined via a plaque forming unit (PFU) assay in LLC-MK2 cells except DENV which, titer was determined with a focus-forming unit assay.

### Virus titration in LLC-MK2 cells

All virus titrations were performed using 12-well standard cell culture plates seeded with cells to reach 100% confluency upon infection. Cells were inoculated with 10-fold serial dilutions of the recovered sample and were rocked at 37°C for one hour after which the inoculum was removed and replaced with an overlay of 1 ml of 1% methyl cellulose (Sigma Catalog # M0512) mixed 1:1 with 2x MEM (20% FBS, 8 mM glutamine, 20 mM NEAA, 2% penicillin/streptomycin, 2 mM sodium pyruvate). Plates were placed in a 37°C incubator with 5% CO2 for 4 days. Cells were stained using 70% ethanol containing 1% wt/vol crystal violet. Plates were incubated for 15 minutes at 22°C after which the fixative was decanted. The plates were rinsed with cold water and dried overnight at room temperature. The titer of DENV was determined through a focus-forming unit assay. Briefly, cells were fixed and permealbilized using 1 ml of a 1:1 acetone/methanol solution with a 60 minute incubation at 4°C. Virus foci were detected using a specific mouse monoclonal antibody from hybridoma 2H2 (Millipore catalog #MAB8705), followed by a horseradish peroxidase-conjugated goat anti-mouse immunoglobulin (Millipore catalog #AP124P), and developed using a 50mg tablet of 3,3’-Diaminobenzadine tetrahydrochloride (Sigma catalog # D5905) dissolved in 20mL PBS with 8uL 30% hydrogen peroxide.

### Infection of cells with viruses

All infections were performed using 24-well standard cell culture plates seeded with cells which, had reached a 90% confluence upon infection. Individual wells were inoculated with 1,000 PFU of virus (≈MOI 0.005) in 150ul of MEM and then rocked at 37°C for one hour after which the inoculum was removed, rinsed twice with sterile PBS, overlaid with 1 ml of DMEM (10% FBS, 4 mM glutamine, 10 mM NEAA, 100mg/ml penicillin/streptomycin, 1mM sodium pyruvate) and incubated at 37°C incubator with 5% CO_2_. Culture supernatants were collected at 1 and 72 hours post-inoculation (PI).

### Visualization of cytopathic effects

All infections were performed using 12-well standard cell culture plates seeded with cells which, had reached a 90% confluence upon infection. Individual wells were inoculated with 1,000 PFU of virus (≈ MOI 0.0025) in 200ul of MEM and then rocked at 37°C for one hour after which the inoculum was removed, rinsed twice with sterile PBS, overlaid with 1 ml of DMEM (10% FBS, 4 mM glutamine, 10 mM NEAA, 100mg/ml penicillin/streptomycin, 1mM sodium pyruvate) and incubated at 37°C incubator with 5% CO_2_. All cell lines were allowed to develop CPE for 7 days PI. Cells were stained using as described earlier. Images were obtained using Micron imaging software (Westover Scientific) and an inverted microscope at 40X magnification.

### Primer design for real-time RT-PCR

Primers for USUV were designed against the USU181 sequence (Genbank accession: JN257984) and amplify a 104 base pair fragment of the envelope protein gene starting at nucleotide position 1325 and ending at position 1428 (Table 2). Primers for ZIKV were designed against the MR766 strain (Genbank accession: AY632535) and amplify a 128 base pair fragment of the envelope glycoprotein starting at nucleotide position 1398 and ending at position 1525. Blasts for these primer sequences showed sequence homology to multiple strains of the respective virus but no homology to other viruses. This protocol did not detect RNA derived from ZIKV strain PRVABC59, a Puerto Rican isolate from the 2015 outbreak. A standard curve for each virus was constructed in which, 10-fold serial dilutions of virus stock that had been titrated in LLC-MK2 cells via PFU assay, were compared to the cycle threshold (Ct) values from the real-time RT-PCR (qRT-PCR). The USUV primer set could detect as few as 10 PFU per mL and the ZIKV primer set was able to detect as few as 100 PFU/mL. Both primer sets did not amplify other arboviruses tested including: WNV, SINV, YFV, DENV serotypes 1–4, and CHIKV. Sequences for the primer sets are listed below:

**Table 2:** Real-time PCR primers used for the detection of USUV and ZIKV.

Virus	Primer Direction	Sequence
**USUV**	Forward	5’-AGCTCTGACACTCACGGCAACTAT-3’
	Reverse	5’-TCACCCATCTTCACAGTGATGGCT-3’
**ZIKV**	Forward	5’-TATCAGTGCATGGCTCCCAGCATA-3’
	Reverse	5’-TCCTAAGCTTCCAAAGCCTCCCAA-3’

### Virus detection via real-time RT-PCR

Of the cell lines tested, only LLC-MK2 cell lines consistently produced viral plaques. The FoLu cell line initially produced large round plaques at 3 days for both ZIKV and USUV but lost the ability to produce plaques after subsequent passaging of the cell line. In order to determine if, and how much, virus was being produced by these cells, qRT-PCR was employed. Viral RNA was extracted from cell culture supernatant using the Ambion MagMax-96 extraction kit (Life Technologies: Grand Island, NY) per manufacturer’s instructions. The qRT-PCR reactions were conducted using a BioRad Superscript One Step SYBR Green qRT-PCR kit (Winooski, VT). The following cycling conditions were employed: reverse transcription at 50°C for 10 min, denaturation at 95°C for 5 min, followed by 40 cycles of denaturation and amplification at 95°C for 10 sec and 55°C for 30 sec. Cycle threshold values were used to estimate relative viral titers of infected cell lines according to a standard curve created using a serial dilution of known viral concentrations of virus that produced plaques in LLC-MK2 cells. Results are expressed as the average of 3 independent trials amplified in duplicate.

A series of controls were included in each plate in order to identify true positives not related to background. A no-template control and a no-primer control were performed to verify that the reagents and equipment were working as expected. A positive virus control was used on each plate. A non-infected cell culture supernatant control was included to verify that there was no increase in non-specific binding from the PCR primers that could cause a higher background signal. Finally, the cell culture supernatants were collected 1 hour PI to ensure that qRT-PCR results, 72 hours PI, were not convoluted by input virus. RT-PCR data were analyzed using the ΔΔCt method. Replicates were pooled, averaged, and standard deviation was calculated. If a standard deviation was greater than 3, any outliers were removed from the analysis. The LLC-MK2 cell line was used as the reference cell line.

### Virus binding assay

To determine if cell resistance to USUV or ZIKV was binding dependent, a virus: cell binding assay was performed as previously described by Thaisomboonsuk, et al [34]. Briefly, confluent LLC-MK2 cells in 6-well plates were rinsed 3 times with ice-cold PBS and then 3 ml of ice-cold binding medium (DMEM containing 0.8% BSA and 25 mM HEPES, pH 6.0) was added to each well. Plates were incubated for 1 hour on ice. The medium was aspirated and 600ul of 10,000 PFU of virus (≈ MOI 0.01) in ice-cold binding medium was added to the cells and incubated on ice for 2 hours with rocking every 15 minutes. The inoculum was then removed and the cells rinsed 3 times with ice-cold PBS. An RNA extraction of the cell monolayers was immediately performed using the Qiagen RNeasy mini kit (Valencia, CA) per the manufacturer’s instructions and qRT-PCR was performed as described above. Results are expressed as the average of 3 independent trials amplified in duplicate.

## Results

### USUV and ZIKV replicate in multiple cell lines

**Fig 1:**
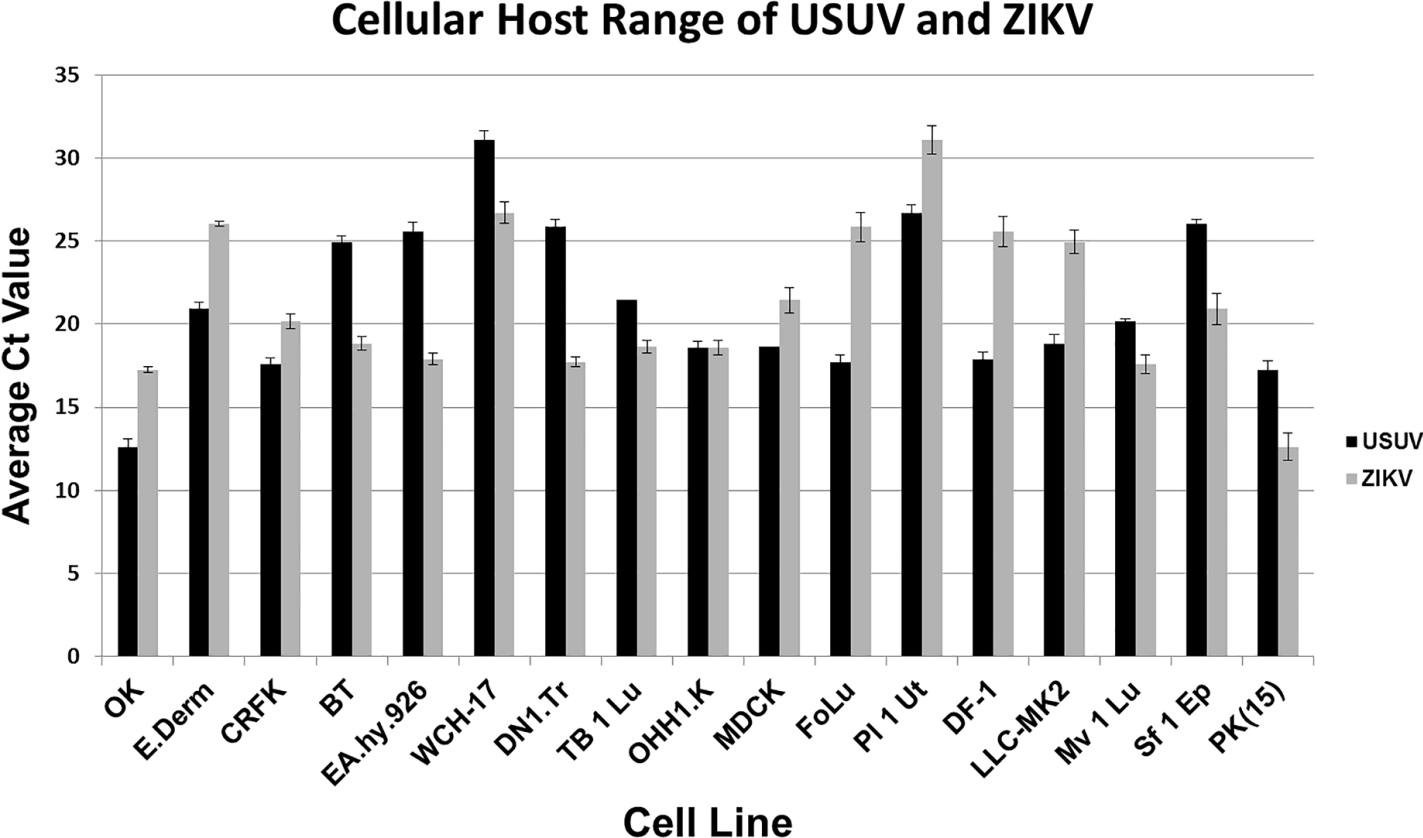
The host range of USUV and ZIKV in cell culture. Average Ct values of USUV and ZIKV ± SEM produced from cell culture supernatants from 17 cell lines collected at 72 hours PI.

Of the 17 cell lines tested, all showed quantifiable Ct values for both USUV and ZIKV based upon qRT-PCR data at 72 hours PI (Fig 1). The cell lines WHC-17, E. Derm, BT, EA.hy.926, Tb 1 Lu, Sf 1 Ep, DNl.Tr, and Pl 1 Ut produced significantly less USUV than the LLC-MK2 cell line (P = 1.27^−07^, 1.71^−03^, 1.4^−08^, 3.97^−07^, 2.48^−04^, 1.84^−06^, 1.7^−08^, 7.64^−08^) (Fig 1). The cell lines OHH1.K, MDCK, FoLu, Mv 1 Lu, and PK(15) were able to replicate USUV as well as the LLC-MK2 cell line (P = 0.66, 0.74, 0.05, 0.09, 0.09) (Fig 1). The OK, CRFK, and DF-1 cell lines were able to produce significantly more USUV than LLC-MK2 cells (P = 2.46^−07^, 0.025, and 0.021) (Fig 1). The cell lines OK, E.Derm, CRFK, OHH1.K, FoLu, and PK(15) produced just as much ZIKV as the LLC-MK2 reference line (P = 0.52, 0.2, 0.16, 0.13, 0.057, and 0.56) (Fig 1). The BT, EA.hy.926, WCH-17, DN1.Tr, Tb 1 Lu, MDCK, Pl 1 Ut, DF-1, Mv 1 Lu, and Sf 1 Ep cell lines produced significantly less ZIKV than the LLC-MK2 line (P = 1.71^−03^, 0.017, 4.67^−08^, 0.038, 1.59^−10^, 6.97^−04^, 1.31^−04^, 2.5^−05^, 7.65^−04^, 8.53^−4^) (Fig 1).

### WCH-17 and Tb 1 Lu cells do not produce significant quantities of ZIKV or USUV

USUV and ZIKV were detected in very low quantities (≈100pfu or less/ml) in WCH-17 cells, over 12-fold less than LLC-MK2 cells (P = 1.27^−07^ and 4.67^−08^). Likewise, Tb 1 Lu cells produced more than 15-fold less ZIKV (≈100pfu or less/ml) than LLC-MK2 cells (P = 1.59^-10^). CPE was not evident for either virus in either cell line. A virus: cell binding assay was performed in order to determine if cell receptors were present that would allow ZIKV or USUV to attach to the Tb 1 Lu or WCH-17 cell surface. The Ct values for all treatments express the amount of virus present in the sample. A Student’s T-test comparing the virus:cell binding of WHC-17 and Tb1. Lu cells to LLC-MK2 cells indicated a lack of difference between the cell lines (Table 3). The statistical similarity of the data suggests that both ZIKV and USUV bind to WCH-17 cells and ZIKV binds to Tb 1 Lu cells as efficiently as they bind to the LLC-MK2 control cells (Table 3).

**Table 3:** ZIKV and USUV bind to Tb 1 Lu and WCH-17 cells. Ct values as determined by qRT-PCR of ZIKV and USUV after binding to LLC-MK2, Tb 1 Lu, and WCH-17 cells.

		Average Ct(±error)	P-value
**ZIKV**	**Tb 1 Lu**	20.57(±0.09)	0.84
**ZIKV**	**WCH-17**	20.19(±0.32)	0.59
**ZIKV**	**LLC-MK2**	20.5(±0.41)	-
**USUV**	**WCH-17**	20.99(±0.12)	0.83
**USUV**	**LLC-MK2**	20.96(±0.01)	-

### USUV and ZIKV produce cytopathic effects in multiple cell lines

**Fig 2:**
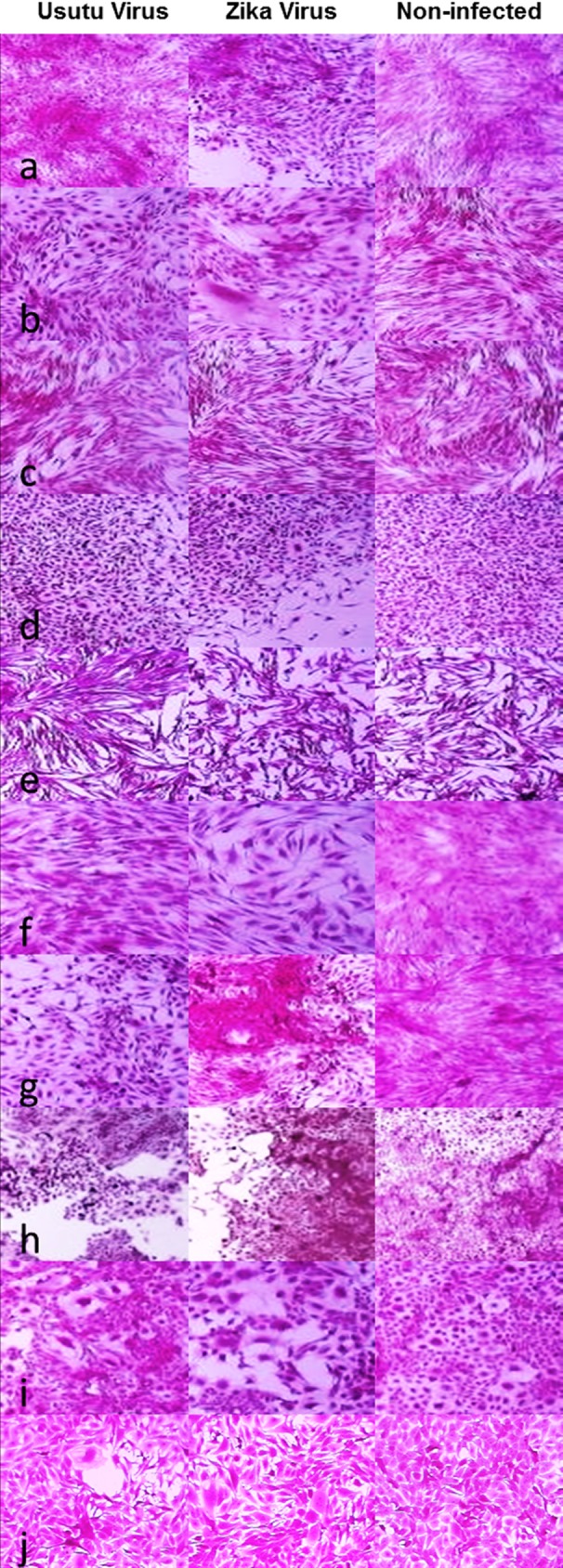
Cytopathic effects of USUV and ZIKV. Cytopathic effects of ZIKV and USUV viruses were visualized a 40X magnification on an inverted microscope. Cytopathic effects were observed for both viruses in CRFK (a), DN1.Tr (b), E. Derm (c), EA.hy.926 (d), FoLu (e), OHH1.K (f), OK (g), PK(15) (h), Sf 1 Ep (i), and Mv 1 Lu (j) cells.

Cytopathic effects were observed in CRFK, DN1.Tr, E. Derm, EA.hy.926, FoLu, OHH1.K, OK, PK(15), Sf 1 Ep, and Mv 1 Lu cell lines from both ZIKV and USUV infection. Forms of CPE caused by USUV in CRFK cells included pyknosis (Fig 2A), while ZIKV caused focal degeneration in addition to pyknosis. Dn1.Tr cells exhibited pyknosis, koilocytes, enlargement, and rounding in response to ZIKV and USUV infection (Fig 2B). E. Derm cells did not show consistent CPE when infected with USUV but did exhibit pyknosis in response to ZIKV (Fig 2C). EA.hy.926 cells produced pyknosis, koilocytes, rounding, and enlargement when infected with ZIKV or USUV however; ZIKV also produced focal degeneration (Fig 2D). FoLu cells produced koilocytes and enlargement when infected with USUV and pyknosis when infected with ZIKV (Fig 2E). USUV produced pyknosis and cellular enlargement in OHH1.K cells and ZIKV produced koilocytes, enlargement and, infrequently, focal degeneration (Fig 2F). OK cells produced pyknosis, koilocytes, enlargement, rounding, and focal degeneration when infected with USUV but only pyknosis and overgrowth when infected with ZIKV (Fig 2G). Focal degeneration, pyknosis, and rounding were produced in PK(15) cells when infected with either USUV or ZIKV (Fig 2H). Sf 1 Ep cells exhibited pyknosis, koilocytes, rounding, and enlargement when infected with either ZIKV or USUV with ZIKV also causing focal degeneration (Fig 2I). Mv 1 Lu cells exhibited focal degeneration and koilocytes in response to USUV infection and cellular enlargement when infected with ZIKV (Fig 2J). LLC-MK2 cells produced focal degeneration and cellular enlargement within 3 days PI and by 7 days PI, most cells were dead and detached (Not pictured).

## Discussion

Though evidence of ZIKV infection has been found in non-primate species, the host range for ZIKV, both *in vitro* and *in vivo*, has not yet been explored. Preliminary studies using the 2001 USUV emergent strain indicated that the virus could infect several species in cell culture [35]. For this experiment, the cellular host range of the prototype ZIKV and USUV isolates was examined in cell lines of various species. Seventeen distinct cell lines were tested including species found only in the Western Hemisphere. Cell lines were selected based on the susceptibility of the host species to flaviviral infection and utility of the cell line in virus research. ZIKV and USUV originated in the Eastern Hemisphere, and as such, have not encountered species like opossums, armadillos, certain cervids, raccoons, as well as foxes and rabbits native to the North and South America. Most of these animals are peridomestic and inhabit the same environment as the mosquito vectors. Mosquito vectors for USUV include various *Culex* and *Aedes* species including *Culex pipiens* and *Aedes albopictus* which, are both found in the Western Hemisphere. ZIKV vectors are limited to *Aedes* species including *Ae*. *aegypti* and *Ae*. *albopictus*. While *Ae*. *aegypti* is considered to be a human-exclusive, several studies have shown that *Ae*. *aegypti*, while preferring humans, will feed opportunistically on cows, goats, birds, pigs, dogs, cats, rats, and horses [36,37]. In the Western Hemisphere, *Ae*. *albopictus* has been shown to feed opportunistically on cows, rats, deer, raccoons, birds, dogs, cats, opossum, pigs, squirrels, mice, and cottontail rabbits [38–40]. *Cx*. *pipiens*, and many other *Culex* species, will feed on humans as well as numerous mammals, birds, and reptiles [41, 42].

We found that both USUV and ZIKV replicate well in cells from many domestic and peridomestic animals. Though not justified by the evidence presented here, these animals may be susceptible to viral transmission through mosquito vectors. The data agree with other work that shows USUV can infect PK (15), MDCK, and primate cells [35] and suggest that the USUV prototype strain may behave similarly in cell culture to the emergent strains of the virus. In addition, this work agrees with recent work showing that DENV can replicate in a variety of cell lines [43, 44]. Unlike ZIKV and USUV, DENV (1–4) exhibited limited infectivity in MDCK, DF-1, Sf 1 Ep, EA.hy.926, BT, and Mv 1 Lu cells [43, 44]. Unfortunately, we were not able to identify other work that described CPE induced by other flaviviuses in these cell lines.

Though ZIKA and USUV did not replicate well in WCH-17 and Tb 1 Lu cells, the data show that ZIKV and USUV bind to WCH-17 cells and ZIKV binds to Tb 1 Lu cells as efficiently as the LLC-MK2 control cells. This suggests that USUV or ZIKV infection of WCH-17 and Tb 1 Lu cells may be inhibited during the virus: cell fusion or the viral replication process. Notably, WCH-17 cells are infected with hepatitis B virus which may be a contributing factor to the inability of these viruses to establish an infection in this cell line [45].

In addition to replicating in various cell lines, USUV induced CPE in 9 of the 17 characterized cell lines. The characteristics of CPE caused by a flavivirus vary in accordance to the host cell [46] and are dependent on various factors including host genetics, viral receptors, immune-response, and defective virus particles [46]. Previous studies on the 2001 USUV emergent strain indicated that CPE was induced in PK (15), Vero, and GEF (goose embryo fibroblast) cells [35]. The range and extent of CPE observed suggests that these cell lines may be useful for virus culture and viral titer studies such as TCID50 and plaque reduction neutralization tests.

Research has shown that USUV is genetically distinct from other flaviviruses [47]. Different strains of USUV have been shown to differ by as much as 5% in amino acid sequence [8]. These amino acid substitutions may influence virulence and other characteristics of USUV [8, 47, 48]. ZIKV too, is genetically distinct from other flaviviruses, and different strains of ZIKV have been shown to differ by as much as 11.7% in nucleotide sequence [19]. Moreover, significant amino acid deletions have been identified at glycosylation sites of the envelope glycoprotein in some strains of ZIKV including the MR 766 strain used in this work [19] which may influence virulence or other characteristics of the virus [49].

USUV and ZIKV may achieve their broad host range by exploiting alternative infectious entry pathways. Cellular membrane components such as clathrin, dynamin, actin, and lipids have been shown to be involved with viral entry into the host cell cytoplasm [50–54]. The impact of these various components on virus entry is has been shown to be host specific for DENV [50] and may be contributing to the ability of USUV or ZIKV to establish infection in a wide variety of cell lines.

### Conclusions

The data herein indicate that several cell lines can be used to culture and study USUV and ZIKV. The susceptibility for certain cell lines to USUV and ZIKV may provide a tool for characterizing these viruses and may provide an *in vitro* platform for the study of host-pathogen interactions, model development, and the development of therapeutics. Additional questions not addressed in this data included whether or not the broad host infectivity observed may be a function of the virus strains that were used for the experiments. These strains may not accurately reflect the characteristics of USUV or ZIKV currently circulating, or that of other laboratory-adapted strains. Finally, the behavior of USUV and ZIKV in the laboratory does not reflect the behavior of these viruses in their natural environment.

## Supporting Information

S1 Data RT-PCR data for cell lines infected with Zika or Usutu virus. The data presented are the raw Ct values and relative viral titers derived from cell culture supernatant collected 72 hours post infection. Relative viral titers were estimated based on a standard curve constructed using serial dilutions of virus which were fit to plaque numbers produced in LLC-MK2 cells.
(XLSX)
